# Real-Life Treatment Outcome of Botulinum Toxin A Injection on Overactive Bladder and Voiding Dysfunction in Patients with Central Nervous System Lesions

**DOI:** 10.3390/toxins16030123

**Published:** 2024-03-01

**Authors:** Yuan-Hong Jiang, Jia-Fong Jhang, Sheng-Fu Chen, Hann-Chorng Kuo

**Affiliations:** Department of Urology, Hualien Tzu Chi Hospital, Buddhist Tzu Chi Medical Foundation, Tzu Chi University, Hualien 970, Taiwan; redeemer1019@yahoo.com.tw (Y.-H.J.); alur1984@hotmail.com (J.-F.J.); madaux@yahoo.com.tw (S.-F.C.)

**Keywords:** lower urinary tract dysfunction, overactive bladder, dysfunctional voiding, neurogenic bladder, botulinum toxin A

## Abstract

Purpose: Neurogenic lower urinary tract dysfunction (NLUTD) is common in patients with neurological lesions in the central nervous system (CNS). Medical treatment usually cannot adequately relieve NLUTD. This study reported the real-life treatment outcome of botulinum toxin A (BoNT-A) for overactive bladders (OAB) and voiding dysfunction in patients with CNS lesions. Methods: We retrospectively analyzed the first-time treatment outcome of 74 patients who received detrusor 100 U BoNT-A for OAB and 45 patients who received a urethral sphincter 100 U BoNT-A injection for voiding dysfunction. The treatment outcome, therapeutic duration, and adverse events (AE) after BoNT-A were compared among different CNS lesions and among patients with different urodynamic characteristics. Results: The study included 74 patients receiving detrusor injections for OAB (36 with cerebrovascular accidents, 13 with Parkinson’s disease, and 25 with dementia) and 45 patients receiving a urethral sphincter injection for voiding dysfunction (26 with cerebrovascular accidents, 7 with Parkinson’s disease, and 12 with dementia). After detrusor BoNT-A treatment, urinary continence was achieved in 28.4% of patients with neurogenic OAB, postoperative difficult urination in 59.5%, acute urinary retention (AUR) in 9.5%, and urinary tract infection (UTI) in 14.9%, with a therapeutic duration of 6.43 months. There were no differences among subgroups or between patients with detrusor overactivity (DO) and DO with detrusor underactivity (DU) in terms of treatment outcomes and AEs. The improvement rate of urethral sphincter BoNT-A injections was 75.6% without any difference among subgroups. After treatment, 24.4% of the patients had exacerbated urinary incontinence, 33.3% had persistent difficult urination, and 15.6% had UTI. Patients with dementia had higher rates of difficult urination and UTI, higher postvoid residual volume, and a shorter therapeutic duration. Patients with DU and those without urethral sphincter dyssynergia had less favorable outcomes after their urethral sphincter BoNT-A injection. Conclusions: The therapeutic efficacy of detrusor BoNT-A injection for OAB due to CNS lesions is limited, with high rates of difficult urination, AUR, and UTI. Although urethral sphincter BoNT-A injection is effective in treating voiding dysfunction; however, exacerbated urinary incontinence and persistent difficult urination remain a problem, particularly in patients with dementia.

## 1. Introduction

Neurogenic lower urinary tract dysfunction (NLUTD) is frequently encountered in many neurological diseases, ranging from cerebrum to peripheral nerves. In patients with neurological diseases, such as cerebrovascular disease (CVA), Parkinson’s disease (PD), and dementia, lower urinary tract symptoms (LUTS) are common sequela. In patients with CVA, PD, or dementia, poor cortical control is specifically associated with inadequate activation of the orbitofrontal cortex, resulting in poor bladder control and incomplete bladder emptying [1]. Therefore, overactive bladder (OAB) symptoms and voiding dysfunction are common LUTS in patients with central nervous system (CNS) lesions [2]. CNS diseases usually occur in elderly patients, with whom urgency urinary incontinence (UUI), reduced bladder sensation, and reduced frontal lobe perfusion are common [3]. These might result in cognitive impairment, reduced bladder fullness sensation, and urinary incontinence [4]. Patients with a CNS lesion, such as CVA and impaired awareness of bladder urgency to void, are likely to have a higher severity of cortical dysfunction [5].

Medical treatment for OAB associated with CNS lesions is not always satisfactory. Therefore, combination therapy using pharmacotherapy and non-pharmacotherapy options provide the best clinical efficacy, as the combination of treatments target different mechanisms of action [6]. For patients with voiding dysfunction due to bladder neck dysfunction or urethral sphincter dyssynergia, an alpha-blocker with baclofen is usually prescribed [7,8]. The adverse events (AE) of postural hypotension after alpha-blocker administration and the general weakness and dizziness after baclofen administration should be monitored in older patients with CNS-related LUTD.

Botulinum toxin A (BoNT-A) has been widely used to treat muscular hypercontractility, and it also modulates sensory function, inflammation, and glandular function [9,10]. Currently, detrusor BoNT-A injection is recommended as the third-line treatment for OAB [11]. The advantages and disadvantages of BoNT-A versus oral OAB medication have been well reported. Detrusor injection of BoNT-A significantly improves OAB symptoms, urodynamic parameters, and quality of life [12]. The disadvantages include decreased bladder fullness sensation, difficulty initiating urination, increased post-void residual (PVR) volume, decreased voiding efficiency (VE), and the risk of acute urinary retention (AUR) and urinary tract infection (UTI) [13]. In some patients who received BoNT-A injections for OAB, especially those who are older, clean intermittent catheterization (CIC) may be necessary [13,14]. 

Voiding dysfunction is common in patients with LUTD due to CNS lesions. Patients with neurogenic lesions and voiding dysfunction often have mixed storage and voiding symptoms [1,2,3]. Patients who are ambulatory and wish to urinate voluntarily without a catheter may prefer resumption of voiding ability [15,16,17]. Previous studies on the effects of urethral sphincter BoNT-A injection on neurogenic voiding dysfunction due to CNS lesions are limited, and the results are controversial.

This study retrospectively analyzed the treatment outcome, AEs, and therapeutic duration of detrusor BoNT-A injection for OAB refractory to medical therapy and urethral sphincter BoNT-A injection for voiding dysfunction in patients with CNS lesions, such as CVA, PD, and dementia. The patients’ overall satisfaction and retreatment rate were also reported.

## 2. Results

Among the patients with CNS lesions, 74 (46 men and 28 women) had OAB refractory to oral medical treatment, and 45 (22 men and 23 women) had voiding dysfunction. The mean age was 68.1 ± 12.8 years (45–88 years) in the OAB group and 69.8 ± 10.5 (42–92 years) in the voiding dysfunction group. The age of patients with CVA and OAB was significantly younger than that of patients with PD and dementia. However, no significant difference of age was noted among patients with CVA, PD, and dementia with voiding dysfunction ().

### 2.1. Detrusor BoNT-A Injection for OAB

An analysis of the therapeutic effects of the first intravesical BoNT-A injections on neurogenic OAB due to CVA, PD, and dementia revealed that the overall dry rate was only 28.4% (21 in 74 patients), while improvement was seen in 54.1% (40 in 74 patients), and failure was seen in 17.6% (13 in 74 patients). The success rate was not significantly different among the three disease subgroups (*p* = 0.894). The treatment outcome was also not significantly different between men and women (*p* = 0.199). After BoNT-A injection, difficult urination was reported in 59.5% of patients, AUR in 9.5%, and UTI in 14.9% (Table 1). Postoperative PVR increased to 44% of bladder capacity, and the therapeutic duration lasted for a mean of 6.4 months. There was no significant difference in the rates of AEs and therapeutic duration among patients with CVA, PD, and dementia. Urodynamic findings, such as DO, DU, and the presence of USD, also did not have an impact on the treatment outcome (Table 2).

### 2.2. Urethral Sphincter BoNT-A Injection for Voiding Dysfunction

In this cohort of 45 patients with CVA (n = 26), PD (n = 7) and dementia (n = 12), the overall rate of improvement in voiding after urethral sphincter BoNT-A injection was 75.6%. The success rate was not significantly different among the three disease subgroups (*p* = 0.347). The treatment outcome was also not significantly different between men and women (*p* = 0.666); however, 24.4% of the patients were bothered by exacerbated UUI, 33.3% still complained of difficult urination, and 15.6% had UTI after a urethral sphincter BoNT-A injection. Patients with dementia had less favorable treatment outcomes, with improvements in only 58.3% of patients, higher rates of difficult urination (83.3%), AUR (16.7%), and UTI (41.7%) after urethral sphincter BoNT-A injection, and a short therapeutic duration (3.17 months) (Table 3). Patients with urodynamic DU had a lower improvement rate than those with DO, whereas patients with urodynamic USD had a better improvement in the postoperative PVR and VE (Table 4).

### 2.3. Satisfaction and Repeated BoNT-A Injections

**Figure 1:**
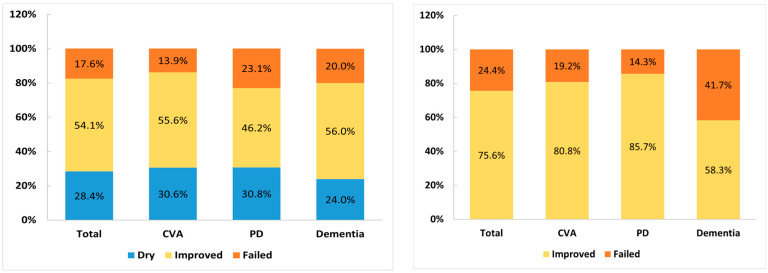
The treatment outcome of detrusor BoNT-A injection for overactive bladder (**left**) and urethral sphincter BoNT-A injection for voiding dysfunction (**right**) among patients with cerebrovascular accident (CVA), Parkinson’s disease (PD), and dementia.

The treatment outcomes of patients with OAB and voiding dysfunction among different CNS lesions are shown in Figure 1. During the follow-up period after detrusor BoNT-A injection for OAB, only 12 of 74 (16.2%) patients continued to receive detrusor BoNT-A treatment more than two times, whereas only 7 of 45 (15.6%) patients received urethral sphincter BoNT-A injections more than two times for voiding dysfunction. The other patients declined further BoNT-A injections mainly because of poor or inadequate therapeutic efficacy, AEs, exacerbated LUTS, and personal decision. Most patients preferred to receive long-term medical treatment rather than continuing BoNT-A injection for NLUTD.

## 3. Discussion

Previous clinical trials have revealed that BoNT-A injection into the detrusor could effectively improve urinary incontinence, and injection into the urethral sphincter could facilitate spontaneous voiding in patients with CNS lesions and NLUTD. This study revealed that the real-life reports of satisfactory rates were low in both lower urinary tract conditions. The continence rate after detrusor BoNT-A injection was only 28.5%, and most patients suffered from difficult urination, AUR, or UTI after treatment. After urethral sphincter BoNT-A injection, 75.6% of patients showed improvement in voiding difficulty; however, exacerbated UUI and persistent difficult urination still bothered these frail, older patients with CNS lesions and ambulatory disturbances. Therefore, only a small proportion of patients with CVA, PD, and dementia received repeated BoNT-A injections.

Patients with CVA, PD, and dementia usually have urodynamic DO and coordinated urethral sphincters, resulting in UUI when bladder capacity is reached. [18] Some patients with CVA may have uninhibited sphincter relaxation due to a frontal lobe lesion, and urethral sphincter dyssynergic activity is common with lesions in the basal ganglia [19]. In older patients with multiple CVAs or lacunar infarctions, DO with DU can be another bladder problem, as patients will experience urgency, UUI, and a large PVR volume [20]. A large proportion of patients with PD present with LUTD, including urgency, increased frequency, and UUI as predominant symptoms [21]. Urodynamic DO was found in 90% of patients with PD and 61% of patients with sporadic involuntary external sphincter activity. Bradykinesia of the urethral sphincter may also cause functional outlet obstruction in PD [22]. Most patients with multiple system atrophy also had a significantly large PVR volume or urinary retention due to impaired detrusor contractility [23]. Patients with dementia and brain lesions at the frontal lobe may have voiding dysfunction and lack of bladder filling sensation. DO, diminished awareness of bladder filling, and inability to suppress the micturition reflex can result in UUI, a large PVR, or urinary retention, depending on the location of the lesion and the severity of brain damage [24]. 

Previous studies of BoNT-A in elderly patients with OAB and in older patients with CNS-related OAB have demonstrated that intravesical injection of 100 U BoNT-A effectively decreased urgency symptoms [9,13,14]. The AEs were acceptable, and the long-term effects were comparable to those in patients with OAB in general. Previous clinical trials also reported that patients with CNS lesions did not experience increased risks of AUR and UTI, but they did experience a higher rate of straining to void [25]. However, the patients enrolled in these clinical trials may have been highly selected. In real-life situations, most patients with CVA, PD, and dementia are usually very old, frail, with low detrusor contractility and an inability to move freely (depending on the severity and subtypes of CNS lesions). In clinical practice, detrusor injection of BoNT-A could not provide a continence result without increases in PVR and difficulty in urination in patients with CNS lesions and OAB. Urethral sphincter injection of BoNT-A could not improve VE without worsening UUI. A balance between the advantages and disadvantages of BoNT-A is difficult to achieve in patients with CVA, PD, and dementia, unless the patient’s condition is mild.

There are few real, randomized controlled clinical trials that investigate the therapeutic efficacy and safety of BoNT-A on OAB due to CNS lesions. Most studies reported a small cohort of patients with CVA, PD, dementia, and other intracranial lesions. There are several reports on the treatment outcome of BoNT-A on neurogenic OAB. Satisfactory outcomes were also noted in patients with PD and OAB [26] and in men with persistent OAB symptoms after transurethral resection of the prostate [27]. Overall, among patients with OAB, older adults who are frail or who have OAB due to chronic CNS disorders can attain the same treatment results as younger patients and older adults without frailty, including significant improvement of OAB symptoms and quality of life. With these considerations, BoNT-A injection into the bladder base may prevent the AEs of difficult urination and urinary retention in frail, older adults with OAB [28].

For patients with neurogenic OAB due to CVA, the most frequent AEs were UTI, difficult urination, and urinary retention after detrusor BoNT-A injection [29]. Suburothelial BoNT-A injection at a dose of 200 U increased bladder capacity and improved the incontinence grade in patients with spinal cord lesions or multiple sclerosis, but only small case series support BoNT-A injection for NDO in patients with PD or CVA [30]. Most researchers would like to manage PD and poststroke patients using BoNT-A 100 U intradetrusor injection to achieve long-term efficacy and reduce adverse effects [26]. After BoNT-A injection, 79.2% of patients with PD and OAB reported an improvement in OAB symptoms, 29.1% experienced resolution of UUI while their PVR increased, and 12.5% of the patients required CIC [31]. Higher preoperative PVR was the strongest predictor of treatment failure and postoperative urinary retention requiring CIC. Among the 16 patients with PD and neurogenic OAB after BoNT-A injection, 20% received a perfect result, 40% experienced significant improvement, 40% failed, and 28% required CIC [32]. A meta-analysis of a randomized controlled trial revealed that, among several interventions for the treatment of neurogenic OAB in patients with PD, there is little or no evidence that treatment improves patient outcomes in this population [33]. 

In this study, we found that patients with dementia had similar therapeutic results from detrusor BoNT-A injections as those in patients with CVA and PD; however, the treatment outcome was inferior in patients with dementia regarding the rates of difficult urination, UTI, and therapeutic duration. Chronic use of OAB anticholinergic medications for more than 3 months is likely associated with an increased risk of new-onset dementia [34]; therefore, BoNT-A detrusor injection has been considered in early dementia patients with normal bladder perception and urinary incontinence. However, the true therapeutic efficacy of BoNT-A treatment is limited in patients with dementia who do not sense bladder urgency or are wheelchair ridden [35]. 

Pilot studies or small case series support BoNT-A for neurogenic DO in patients with PD and CVA [30]. A study reported that BoNT-A 100 U injection in PD with UUI revealed moderate to marked symptom relief at 3 months and a 50% incontinence decrease over a 6-month period [36]. In another study of 140 patients with neurogenic OAB, the 10-year discontinuation-free and failure-free survival rates were 49.1% and 73%, respectively. Causes of BoNT-A discontinuation included treatment failure (43.7%), patient decision (28.1%), improvement of OAB (14.1%), neurological progression (12.5%), and AEs (1.6%) [37]. Our study showed that persistency in BoNT-A injection was low for OAB or voiding dysfunction in patients with CVA, PD, and dementia. The results further reflect a discrepancy of treatment outcomes between clinical practice and clinical study.

Regarding the influence of urodynamic characteristics on the treatment outcome of OAB in patients with CNS lesions, DO with and without DU did not affect the success rate and AEs after detrusor BoNT-A injection. It is likely that most patients could not achieve continence after BoNT-A detrusor injections and that most patients would develop an underactive bladder after this treatment. Instead, patients with DU and non-USD experienced a lower benefit from urethral BoNT-A injection for voiding dysfunction. This result indicates that BoNT-A can only be effective in patients with a hyperactive urethral sphincter but could not improve voiding efficiency simply by reducing urethral resistance. Although urethral sphincter BoNT-A injection is effective in improving VE, incomplete bladder emptying remains a problem in patients with CNS lesions who have impaired awareness of bladder sensation [38]. Therefore, patients with neurogenic voiding dysfunction might not be satisfied with the therapeutic results of a urethral sphincter BoNT-A injection.

Among the patients with OAB due to CVA, PD, or dementia, patients with PD had significantly less PVR than patients with CVA or dementia after detrusor BoNT-A injection, while patients with dementia had a relatively shorter therapeutic duration. Regarding urethral sphincter BoNT-A injection for voiding dysfunction, significantly higher rates of difficult urination, UTI, short therapeutic duration, and relatively lower VE were noted in patients with dementia. The poor treatment outcome in dementia patients could be due to poor cortical cognitive and perceptive function, as well as low detrusor contractility.

The results of this study provide real-life therapeutic outcomes for BoNT-A detrusor or urethral sphincter injections in patients with OAB or voiding dysfunction due to CNS lesions. BoNT-A has been licensed and widely used in patients with OAB due to idiopathic DO. However, the use of BoNT-A in NLUTD due to CNS lesions has not been well documented. Patients with CVA, PD, or dementia usually have both storage and emptying LUTD because of poor cortical control, low detrusor contractility, and urethral sphincter dysfunction. Therefore, these patients may more easily to develop unexpected adverse events after BoNT-A detrusor or urethral sphincter injection. The undesired adverse events, such as difficulty in urination, large PVR, need of CIC, and risk of UTI, might be bothersome issues after intravesical BoNT-A injections, which cause a low rate of repeat BoNT-A injections. In selecting patients with CNS lesions for BoNT-A injection, a low detrusor contractility might predict a poor treatment outcome. The advantages and disadvantages after a BoNT-A injection should be thoroughly informed, and CIC after detrusor BoNT-A injections should be explained well to the patients or caregivers.

A limitation of this study is the unbalanced case numbers among patient groups. Because this study is a retrospective analysis of our experience of BoNT-A injection in patients with OAB and voiding dysfunction due to CNS lesions, the case numbers were not balanced among disease subgroups, or between urodynamic subgroups, which may result in unreliable statistical results.

## 4. Conclusions

NLUTD due to CNS lesions such as CVA, PD, and dementia is common. LUTD involves storage and emptying problems. BoNT-A intravesical injections provide an opportunity to resume urinary continence, and urethral sphincter BoNT-A injections can improve VE. However, undesired AEs, such as difficulty in urination, a large PVR, CIC requirement, and risk of UTI, might be bothersome issues after detrusor BoNT-A injections. Exacerbated UUI, persistent difficult urination, and incomplete bladder emptying may limit the therapeutic application of BoNT-A in patients with NLUTD and CNS lesions. Patients who are refractory to medical treatment and are interested in BoNT-A injection to improve LUTS should be informed of the potential AEs before treatment initiation.

## 5. Materials and Methods

A total of 119 patients with CVA (n = 62), PD (n = 20), and dementia (n = 37) were retrospectively analyzed for the therapeutic efficacy, AEs, therapeutic duration, and persistency in BoNT-A injection for NLUTD. Detrusor injection of 100 U BoNT-A was performed in 74 patients (36 with CVA, 13 with PD, and 25 with dementia) for OAB, and a urethral sphincter injection of 100 U BoNT-A was performed in 45 patients (26 with CVA, 7 with PD, and 12 with dementia). All patients underwent a videourodynamic study before BoNT-A injection to confirm their bladder and urethral dysfunctions, such as detrusor overactivity (DO), detrusor underactivity (DU), bladder neck dysfunction, or urethral sphincter dyssynergia (USD). Patients with LUTD due to benign prostatic hyperplasia (BPH) were not included in this study.

All patients had received medical treatment for NLUTD for at least 6 months before BoNT-A injection. Patients with OAB had been treated with antimuscarinic agents, beta-3 adrenoceptor agonists, or combined medication; however, the OAB symptoms still persisted [39]. Patients with CNS lesions and voiding dysfunction had been initially treated with alpha-blockers, skeletal muscle relaxants, and 5-alpha-reductase inhibitors if there is evidence of BPH, but the patients were still bothered by difficult urination, large PVR, or chronic urinary retention.

Detrusor BoNT-A injections were performed, as previously reported [25]. In brief, patients received 20 detrusor injections of 100 U onabotulinumtoxinA (BOTOX, Allergan, Irvine, CA, USA) at the posterior and lateral walls of the bladder, and 5 U of BoNT-A in 0.5 mL of normal saline was injected at each site. Urethral sphincter injections were performed by injecting 100 U of BoNT-A at five sites along the urethral wall at an injection depth of 1.5–2.0 cm. Each injection contained 20 U in 0.5 mL of normal saline [39]. The patients were followed up at an outpatient clinic after BoNT-A injection for voiding efficiency, PVR volume, persistent or de novo LUTS, and episodes of UTI. The treatment outcome of detrusor BoNT-A injection for OAB was assessed as follows: dry, completely continent; improved, decrease in episodes of urinary incontinence; and failed, no change in incontinence. The treatment outcome of urethral sphincter BoNT-A injection was classified as improved (less difficulty in urination) and failed (no change in difficulty in urination). Patients were questioned on their satisfaction regarding their BoNT-A injection and their willingness to repeat BoNT-A injection for NLUTD.

The treatment outcome, AEs, and therapeutic duration of detrusor BoNT-A injection for OAB and urethral sphincter BoNT-A injection for voiding dysfunction were compared between different CNS disease subgroups, as well as between patients with different urodynamic characteristics. Differences among groups were analyzed using the chi-square test for categorical variables or the analysis of variance test for continuous variables. All tests were two-sided, and *p* values ≤ 0.05 were considered statistically significant. Statistical analyses were performed using IBM SPSS Statistics, v26. The patients’ data collected in this retrospective study was approved by the Buddhist Tzu Chi General Hospital Research Ethics Committee (IRB: 104-23-A, and 105-151-B). Although most of the data were very old, in this study we traced from the chart for the patients who received repeat BoNT-A treatment after their first treatment. Due to the nature of retrospective analysis, the informed consent form was waived.

## Figures and Tables

**Table 1:** Therapeutic efficacy, adverse events, and therapeutic duration of detrusor BoNT-A injection for patients with CNS lesions and OAB.

Treatment Outcome	CVA (*n* = 36)	PD (*n* = 13)	Dementia (*n* = 25)	Total (*n* = 74)	*p* Value
Age	61.2 ± 14.2	77.8 ± 5.8	73.1 ± 6.6	68.1 ± 12.8	0.000
Dry	11 (30.6%)	4 (30.8%)	6 (24.0%)	21 (28.4%)	0.894
Improved	20 (55.6%)	6 (46.2%)	14 (56.0%)	40 (54.1%)	
Failed	5 (13.9%)	3 (23.1%)	5 (20.0%)	13 (17.6%)	
Difficult urination	24 (66.7%)	5 (38.5%)	15 (60%)	44 (59.5%)	0.206
AUR	5 (13.9%)	0	2 (8.0%)	7 (9.5%)	0.454
UTI	8 (22.2%)	0	3 (12.0%)	11 (14.9%)	0.171
PVR (% CBC)	48.7 ± 23.5	30.4 ± 17.1	45.2 ± 23.3	44.3 ± 23.2	0.047
VE (% CBC)	51.3 ± 23.6	68.9 ± 18.7	54.8 ± 23.3	55.5 ± 23.3	0.063
Duration (mean ± SD)	7.72 ± 6.16	6.46 ± 6.46	4.56 ± 3.69	6.43 ± 5.62	0.095

CVA: cerebrovascular accident, PD: Parkinson’s disease, AUR: acute urinary retention, UTI: urinary tract infection, PVR: post-void residual, VE: voiding efficiency, CBC: cystometric bladder capacity.

**Table 2:** Therapeutic efficacy, adverse events, and therapeutic duration of detrusor BoNT-A injection for patients with CNS lesions and different urodynamic characteristics.

	DO (n = 66)	DO + DU (n = 8)	*p* Value	USD (n = 6)	Non-USD (n = 68)	*p* Value
Dry	21 (31.8%)	0	0.060	1 (16.7%)	20 (29.4%)	0.529
Improved	35 (53.0%)	5 (62.5%)		5 (83.3%)	35 (51.5%)	
Failed	10 (15.2%)	3 (37.5%)		0	13 (19.1%)	
Difficult urination	37 (56.1%)	7 (87.5%)	0.132	4 (66.7%)	40 (58.8%)	1.000
AUR	5 (7.6%)	2 (25.0%)	0.163	0	7 (10.3%)	1.000
UTI	9 (13.6%)	2 (25.0%)	0.339	0	11 (16.2%)	0.583
PVR (% CBC)	44.4 ± 23.0	43.8 ± 26.2	0.946	43.3 ± 17.8	44.4 ± 23.7	0.917
VE (% CBC)	55.5 ± 23.2	56.3 ± 26.2	0.928	56.7 ± 17.8	55.4 ± 23.8	0.903
Duration (mean ± SD)	6.80 ± 5.78	3.38 ± 2.50	0.103	7.33 ± 1.51	6.35 ± 5.84	0.685

DO: detrusor overactivity, DU: detrusor underactivity, USD; urethral sphincter dyssynergia, AUR: acute urinary retention, UTI: urinary tract infection, PVR: post-void residual, VE: voiding efficiency, CBC: cystometric bladder capacity.

**Table 3:** Therapeutic efficacy, adverse events, and therapeutic duration of urethral sphincter BoNT-A injection for patients with CNS lesions and voiding dysfunction.

Treatment Outcome	CVA (n = 26)	PD (n = 7)	Dementia (n = 12)	Total (n = 45)	*p* Value
Age	68.9 ± 11.7	71.6 ± 11.8	70.7 ± 6.8	69.8 ± 10.5	0.793
Improved	21 (80.8%)	6 (85.7%)	7 (58.3%)	34 (75.6%)	0.347
Failed	5 (19.2%)	1 (14.3%)	5 (41.7%)	11 (24.4%)	
UUI	6 (23.1%)	3 (42.9%)	2 (16.7%)	11 (24.4%)	0.489
Difficult urination	3 (11.5%)	2 (28.6%)	10 (83.3%)	15 (33.3%)	0.000
AUR	0	0	2 (16.7%)	2(4.4%)	0.088
UTI	0	2 (28.6%)	5(41.7%)	7(15.6%)	0.001
PVR (% CBC)	32.5 ± 23.0	40.0 ± 27.7	55.4 ± 31.1	39.8 ± 27.3	0.052
VE (% CBC)	67.5 ± 23.0	60.0 ± 27.7	44.5 ± 31.1	60.2 ± 27.3	0.052
Duration (mean ± SD)	5.58 ± 4.71	10.3 ± 7.52	3.17 ± 3.10	5.67 ± 5.27	0.014

CVA: cerebrovascular accident, PD: Parkinson’s disease, AUR: acute urinary retention, UTI: urinary tract infection, PVR: post-void residual, VE: voiding efficiency, CBC: cystometric bladder capacity.

**Table 4:** Therapeutic efficacy, adverse events, and therapeutic duration of urethral sphincter BoNT-A injection for patients with CNS lesions and different urodynamic characteristics.

	DO (n = 41)	DU (n = 4)	*p* Value	USD (n = 43)	Non-USD (n = 2)	*p* Value
Improved (n = 34)	33(80.5%)	1 (25.0%)	0.040	34(79.1%)	0	0.056
Failed (n = 11)	8 (19.5%)	3 (75.0%)		9(20.9%)	2(100%)	
UUI	11 (26.8%)	0	0.558	11 (25.6%)	0	1.000
Difficult uriantion	15 (36.6%)	0	0.285	13 (30.2%)	2 (100%)	0.106
AUR	2 (4.9%)	0	1.000	1 (2.3%)	1 (50.0%)	0.088
UTI	7 (17.1%)	0	1.000	6 (14.0%)	1 (50.0%)	0.290
PVR (% CBC)	39.5 ± 28.0	42.5 ± 22.2	0.837	37.3 ± 25.3	92.5 ± 3.53	0.004
VE (% CBC)	60.5 ± 28.0	57.5 ± 22.2	0.837	62.7 ± 25.3	7.50 ± 3.54	0.004
Duration (mean ± SD)	5.93 ± 5.21	3.00 ± 6.00	0.295	5.93 ± 5.25	3.00 ± 6.00	0.121

DO: detrusor overactivity, DU: detrusor underactivity, USD; urethral sphincter dyssynergia, AUR: acute urinary retention, UTI: urinary tract infection, PVR: post-void residual, VE: voiding efficiency, CBC: cystometric bladder capacity.

## Data Availability

The data used in this study are available upon the reader’s request, after approval by the Ethics Committee of the institution.
